# “*My Heart and My Brain Is What's Bleeding, These Are Just Cuts*.” An Interpretative Phenomenological Analysis of Young Women's Experiences of Self-Harm

**DOI:** 10.3389/fpsyt.2022.914109

**Published:** 2022-07-14

**Authors:** Hilary Norman, Lisa Marzano, Andrea Oskis, Mark Coulson

**Affiliations:** ^1^Faculty of Science and Technology, Middlesex University, London, United Kingdom; ^2^School of Psychology, Faculty of Social Sciences, University of East Anglia, Norwich, United Kingdom

**Keywords:** self-harm, interpretative phenomenological analysis (IPA), stigma, qualitative, suicide

## Abstract

Engagement in self-harm, defined as intentional self-poisoning or self-injury irrespective of the apparent purpose of the act, is increasing, particularly among girls and young women. Understanding the behavior from the perspective of those who self-harm is, therefore, vital in designing effective interventions and treatments. The current brief research report presents a key theme from an Interpretative Phenomenological Analysis of the experience of self-harm among eight young women, aged between 18 and 29. The theme *Is Self-Harm Bad?* concerns the way in which participants both acknowledged and resisted a negative conception of self-harm that was often constructed from other people's attitudes. Three subthemes explore the reasons why participants were reluctant to endorse self-harm as bad: Self-Harm is the Symptom, Self-Harm Works (Until it Doesn't) and Self-Harm is Part of Me. The findings highlight the disparity between the characterization of self-harm as a highly risky behavior and the lived experience of self-harm as a functional means of emotion regulation. From a clinical perspective, the findings explored in this brief report suggest that highlighting the risks of self-harm may not be a sufficient deterrent. The recently revised draft National Institute for Health and Care Excellence (NICE) guidance recommends that everyone presenting to hospital following self-harm should be given a comprehensive psychosocial assessment, of which the function is, in part, to understand why the person has self-harmed. The current study underlines the importance of seeing past the behavior to the underlying causes and exploring the meaning of self-harm to the individual in order to implement effective preventative interventions.

## Introduction

Self-harm, defined as intentional self-poisoning or self-injury irrespective of the apparent purpose of the act (1), carries risks for the individual, including a significantly higher chance of subsequently dying by suicide (2). These risks lie behind the clinical imperative to “*reduce recurrence*” of self-harming behaviors (p. 1) (1). However, such efforts may be hampered by different conceptualisations of self-harm by clinicians (and other potential help-givers) and the individuals who engage in self-harm. While clinicians may see self-harm as a maladaptive and risky behavior, those who engage in it may see it as a “*necessary pain”* (p. 154) (3), and a vital way of coping with otherwise intolerable distress (4).

The failure to appreciate these different perspectives can have important consequences. Individuals who self-harm have been described as manipulative or impossible to help, leading to frustration and a lack of empathy in front-line medical staff (5). Emotional or angry reactions by parents to their children's self-harm may increase feelings of guilt and distress, resulting in further self-harm (6). Negative responses such as these to self-harm disclosure (or the anticipation of them) can affect an individual's willingness to seek help and lead to self-harm being carried out in secret (7, 8).

In their benefits and barriers model, Hooley and Franklin identify social norms, and specifically the cultural non-acceptability of self-harm, as one of five factors that dissuade most people from engaging in self-harm (9). They describe how people who self-harm may bypass the social norms barrier by hiding the behavior from others, or by finding a different set of norms among a group of people who also self-harm. However, even if such measures spare the individual from the approbation of others, the awareness that self-harm is viewed negatively by society still persists. Qualitative research can provide an insight into how the widely-held, negative view of self-harm affects those who engage in it. The current brief research report presents new evidence from an Interpretative Phenomenological Analysis (IPA) of the experience of self-harm among young women who reported difficulty identifying and describing feelings. Two themes from this study have been published elsewhere (10). In this report, we focus on the way in which the participants' experience of self-harm fitted, or conflicted with, the idea that it was a maladaptive, unhealthy way of coping.

## Method

The method for this study was described in Norman et al. (10). In brief, eight women, aged between 18 and 29 (*M* = 22, *SD* = 4.14), were recruited from Middlesex University and the general public, having taken part in an online survey about self-harm. IPA studies are commonly based on a small number of participants, to allow an in-depth analysis of each case (11). The main study focussed on self-harm in people who had difficulties identifying and expressing feelings, and therefore the inclusion criteria required that participants scored above 51 on the Toronto Alexithymia Scale (TAS20) (12). Additionally, to focus on recent experiences, all participants had self-harmed within the past five years (three within the past year). Four semi-structured interviews were conducted in person, three took place online via Skype and a further one via Skype messenger at the participant's request. The interviews were carried by the lead author, a Samaritan listening volunteer. They opened with a broad question, asking participants about their experience of self-harm. Follow-up questions and prompts encouraged participants to elaborate on their feelings in relation to self-harm, both at the time and in retrospect. The spoken interviews ranged from 49 min to 1 h 40 min (average 71 min). Due to connection issues, the interview conducted via Skype messenger took 4 hours, 13 min.

The study was granted ethical approval by Middlesex University Ethics Committee (reference 4083). Steps were taken to ensure the participants' wellbeing, including the collaborative drawing up of a safety plan, and the use of a Visual Analogue Scale (VAS) at the start and end of the interview to gauge the impact on mood (13). Participants were fully briefed about the nature of the study and the voluntary nature of their participation, and provided written or oral (recorded) consent.

Interpretative phenomenological analysis was chosen because it is a phenomenological method focussed on participants' subjective experience, while acknowledging the interpretative role of the researcher in the analytic process (11). The interviews were transcribed verbatim. Following several readings, each transcript was analyzed separately to identify descriptive, linguistic and conceptual comments (11). Emergent themes were developed and then combined into subthemes and super-ordinate themes, which were then compared and combined across the dataset. These stages were carried out by the lead author; the second author independently reviewed one transcript. The lead author made reflexive notes throughout the period of data collection and analysis to aid reflection on the interpretative process.

Four themes were identified: The Obscure Self; Words Fail Me; Control and Compulsion; and Is Self-Harm Bad? The first two themes were presented in Norman et al. (10). This current brief report focuses on the last of the four themes: *Is self-harm bad?*

## Findings

### Self-Harm Is Bad But…

This theme explores participants' feelings toward self-harm, in particular the way in which they both acknowledged and resisted the social construct of self-harm as “bad.”

All the participants expressed, either explicitly or implicitly, the view that self-harm is an unhealthy, negative behavior. At the start of each interview, participants were asked a very general question about their experiences of self-harm.

P3: “*Well I think I started self-harming when I was 17 in high school and [pause] I it got it was real bad for about two years and I would do the whole you know we're gonna we're gonna stop doing this because it's bad and my best friend hates that I do it, and then keep doing it.”*

The word “bad” is used twice in this short extract, first to describe the seriousness of her engagement in self-harm and second as a reason why she felt she ought to stop. The perception of self-harm as bad was endorsed, or even formed, by the reaction of her best friend, whose judgment she presumably valued. An experience shared by the participants was that friends, parents and health practitioners often (although not exclusively) responded unfavorably to self-harm, creating a negative construct against which participants had to position themselves. For example, six participants described having to manage other people's reactions to their scars. Here, P1 remembered going out with her boyfriend and other friends.

P1: “*When I got to the pub I sat at the table and I started taking* [my cardigan] *off and my boyfriend was “No you can't take that off”.”*
I: “*How did you feel about that?”*
P1: “*And I was just sort of I don't know it was like a punch in the stomach. Um cos then you're then sort of like not only me feeling really embarrassed and inadequate but thinking oh my god he feels embarrassed about me, like he's embarrassed to be with this person who's got these scars so and so and I sort of immediately went back into my shell and did my not talking to anyone kind of face and I think a few minutes later he sort of I think he realized that what he said wasn't appropriate and he apologized and he was like no no no you can do whatever you want*”

Her boyfriend's instinctive reaction to P1 revealing her scars in public, and in front of friends, suggested that he saw the scars as embarrassing or even shameful. P1 inferred that he was ashamed, not only of the scars, but of her as a person. Her first reaction was to hide both her scars and also her own feelings. Such negative encounters caused participants to feel guilty, which sometimes increased their recourse to self-harm.

P4: “*Then after it would be like guilt for doing it. But then you feel like you need to punish yourself more because you punished yourself in the first place.”*

Another consequence of other people's reactions was to deter participants from seeking help. P6 described how she had felt better able to manage self-harm safely before other people found out about it.

P6: “*There wasn't any reason to tell anyone. there was a lot of reasons not to tell anyone. I think to be honest, that was the time with the least risk and virtually no escalation because it didn't include anyone else's thinking, questioning, understanding, misinterpretation, stereotypes or pressures.”*

However, while the construct of self-harm as ‘bad' was acknowledged by participants, it was also resisted. For example, in the extract above, P1 appeared to push back against her boyfriend's initial reaction to her scars, describing his response as “*not appropriate*”. Having observed this conflict in how participants viewed self-harm, we identified reasons why it might occur. Three subthemes were identified.

### Self-Harm Is a Symptom

The first reason why participants appeared to resist the idea of self-harm as ‘bad' was that they viewed it as a symptom of underlying mental health difficulties or life stresses. It was necessary to look beyond the behavior to the distress that it signaled.

P8: “*Like I wrote a song, like and it was just like, like “you say I should stop, I shouldn't do this to myself. You say you've had enough. You can't help if I don't want the help. Do you not see I just don't need it. Really, my heart and my brain is what's bleeding, these these these are just cuts*.”

Through her song P8 expressed her frustration that people appeared unable to see past her cuts to the pain underneath. She herself downplayed the significance of the cuts. To her they were an external manifestation of the internal ‘bleeding'. Constructing self-harm as the problem appeared to give other people permission to absolve themselves of any responsibility for her distress. They blamed her for choosing to self-harm and placed the onus for her recovery onto her (“*You can't help if I don't want the help*”). In another example, the participant described how self-harming behaviors caused clinicians to jump to an immediate and, in her view, unhelpful diagnosis.

P1: “*There's a bit of a tendency at the moment when someone's self-harmed once, they immediately have emotional unstable personality disorder and they don't care about the other symptoms at all and I'm kind of like and whenever that happens then there's all the trouble, there's the medic- needs medication doesn't need medication blah blah blah”*

### Self-Harm Works (Until It Doesn't)

The second reason why participants appeared to resist the construction of self-harm as ‘bad' is because it worked for them. All described self-harm as a means of coping and, to a varying extent, necessary to them at certain times in their lives. Self-harm was used to manage an emotional experience that was overwhelming or difficult to understand.

P4*: “It felt like it was a relief for me, I don't know if it was like, it gave me the ability to feel something other than just sadness*.”
P7: “*It kind of just made me forget, and make me focus about, on something else, because when I cut I focussed on that, and also the process after cutting*.”

At the extreme, two participants explicitly described how they felt it saved them from taking their own lives. For P5 self-harm was bad but not as bad as killing herself. She credited self-harm for suppressing suicidal thoughts.

P5: “*It keeps me alive to a certain degree […] and if I have to decide between self-harm and suicide, um self-harm is the lesser of two evils, and I have to say, when I'm not psychotic and when I can actually think things through rationally, self-harm is a good way to calm down suicidal thoughts, it's a compensation, and if I can self-harm and not kill myself and I don't know any other way not to kill myself then in my mind, like self-harm is better than me trying to kill myself in a way*.”

However, the same participant acknowledged that at times this ‘rational' logic would break down and self-harm would not be sufficient to protect her from potentially lethal actions.

P5: “*It will come to a point when my mind set turn to I'll do whatever to myself and I don't care whether that will kill me or not. […] It does work to a point that it doesn't.”*

### Self-Harm Is Part of My Story

The third reason why participants appeared to feel conflicted about the idea of self-harm as ‘bad' lay in the role it played in their own narratives. If they were to acknowledge the social construct of self-harm as bad, participants would in effect be implying that they, as people who had self-harmed, were also bad. At the time of their self-harm, that was, indeed, how they sometimes felt.

P5: “*It gives a perfect reason for why self-harm is the right thing to do, because if I'm a bad person then I deserve that pain and that sort of state of mind and everything that comes with it.”*

In contrast, the five participants whose last self-harm had occurred over a year ago, expressed greater acceptance of their past behavior. Their reflections often revealed a complex mix of feelings, as illustrated in this extract from P2's interview:

I: “*So, how would you say self-harm, if you would, has self-harm affected your life?”*
P2: “*I thought about that, and I still don't know. All I know is it was a big part of my life and who I was for a really long time and it shaped me into the person that I am today but at the same time that I'm glad that I don't do it anymore, and I hope that I never do do it again, um but I think ultimately considering the end product, where I am now, I think it was, [sigh] I can't say that, I want to say that it was a good thing because it kind of ended up in me getting help from my parents and talking to them about it and I don't know what the alternative would have been if I never, if I never did it. So I'm hesitant to say that I think it was a good thing because it brought me closer to my friends and my family.”*

P2, and three other participants, explicitly articulated their belief that self-harm had shaped the people they had become. This person (the “*end product*”) was someone P2 was proud to be and therefore she could not write off self-harm as wholly negative. Here, P2 focussed on the benefit self-harm brought to her which was ultimately to make her closer to the people around her. Other participants argued that self-harm had made them more empathetic, particularly with people going through similar experiences. At the same time, P2 acknowledged that she was glad that she no longer self-harmed, and she was reluctant fully to endorse self-harm as a positive experience (“*I'm hesitant to say*..”). This extract appears to illustrates P2's attempts to create a narrative which gives meaning to her own story whilst acknowledging the accepted view of self-harm as an unhealthy behavior.

## Discussion

Through the identification of conflicting feelings held by the young adult participants about their self-harm, the current study extends our understanding about the subjective experience of this behavior. Participants both acknowledged and resisted the idea that self-harm was “bad.” Three reasons for this resistance were identified: first, that self-harm was a symptom of underlying problems; second, that self-harm worked and served a useful function for participants; and third, that self-harm was an integral part of their personal narratives, which had contributed to the people they had become. The findings highlight the disparity between the characterization of self-harm as a highly risky behavior (14) and the lived experience of self-harm as a functional means of emotion regulation.

The analysis revealed how participants had to position their self-harm in the context of other people's, often negative, views. Many studies have highlighted similar stigmatizing responses to self-harm (15), including in medical settings (16, 17). The fear of stigmatized reactions can have serious consequences for help-seeking (18). For example, one study found that the perceived distinction between “genuine” self-harm and people who were “attention seeking” appeared to increase individuals' propensity to self-harm in secret and to avoid asking for help (19).

Other people's negative perceptions of self-harm may also contribute to the individual's sense of guilt, leaving them “*trapped in a maintenance cycle of shame and self-injury”* (p. 58) (20). This idea is captured in the experiential avoidance model of self-harm, which proposes that self-harm may be maintained in part by the desire to avoid the negative feelings of remorse that may arise as a result of the act itself (21). This cycle was evident in the accounts given by the participants in the current study. However, although other people's negative views of self-harm led to feelings of guilt and shame, they were also resisted, for the reasons identified in the three subthemes.

The first subtheme described how participants felt self-harm was not in itself “bad,” but rather was the symptom of underlying problems. Similarly, participants in Rayner and Warne's study (20) identified the need for medical staff to validate the individual rather than focus only on their self-harming behaviors. Elsewhere, the outcome measures conventionally used in trials of treatments for self-harm, such as a reduction in the frequency of self-harm or lower engagement with services have been criticized by participants (22). Such measures were rejected in part because they dealt only with the symptom of self-harm rather than the psychological or contextual issues, and failed to consider what “recovery” might look like to the individual.

The second reason why participants appeared to resist the idea of self-harm as bad was that it worked for them. The functions served by self-harm for participants mirrored those identified in the wider literature, particularly regarding affect regulation and self-punishment (23–25). The anti-suicide function of self-harm has also been observed in other studies, including in both community (26) and clinical (27) adolescent samples. Harris (28) identified how self-harm has an ‘internal logic' for those who engage in it, which medical professionals, who may view self-harm as an irrational behavior, can struggle to understand.

Conceptualizing self-harm as bad, therefore, may alienate people who feel it serves a unique and useful function in their lives, enabling them to cope with difficult emotional experiences and, at an extreme, helping them avoid suicidal behavior.

Nevertheless, there is considerable evidence that self-harm of any kind is one of the highest risk factors for subsequent death by suicide (29–31). It also carries risks of scarring or organ damage. Evidence from the current study and elsewhere shows that individuals who self-harm are not oblivious of the risks (3, 8). However, Woodley et al. (8) identified a cognitive dissonance in the way their participants held apparently contradictory beliefs about the dangers and benefits of self-harm, that, at times, led them to downplay the risks. Participants in the current study also described how they sometimes self-harmed without knowing whether they wanted to live or die – an ambivalence which has been observed in other studies (32).

The third subtheme suggested that participants were reluctant to condemn self-harm as bad in order not to condemn their past selves. Viewed with hindsight, self-harm had become part of their story. These reflections appear to chime with the idea that self-harm may coincide with developmental challenges in adolescence that begin to resolve in adulthood (33). Other qualitative accounts have similarly highlighted the way people make sense of self-harm as a formative experience within their personal narrative (34, 35). Sutherland et al. (36) found that an attitude of acceptance and self-compassion was helpful in the process of recovery. This may explain the contrast in the current study between the guilt participants felt at the time self-harmed and the more benevolent feelings about past behaviors. Adopting an attitude of acceptance may be fundamental to enabling individuals to stop self-harming and to find other ways of coping.

## Implications

The new UK draft guidelines on the assessment, management and preventing reoccurrence of self-harm (1) underline the importance of conducting a psychosocial assessment after an incident of self-harm, in order to “*develop a collaborative therapeutic relationship with the person”* and to “*begin to develop a shared understanding of why the person has self-harmed*.” (p. 11) (1). The current study provides strong support for this objective, in particular the need to look beyond the behaviors to the underlying individual and environmental factors, and to understand the function played by self-harm. Demonstrating an understanding of the meaning of self-harm to the individual can encourage help-seeking (6) and may be essential in the process of stopping (37). For the guidance to be put into effective practice, training and support will be needed for those who come into contact with people who have self-harmed, in both clinical and non-clinical settings, to ensure that disclosure experiences are positive and not alienating (16). Even brief training programmes have been shown to be effective in changing attitudes toward people who self-harm (38).

This study also suggests that care is needed in the way in which the risks of self-harm are communicated to people who engage in it. In the subtheme, *Self-Harm Works*, participants described the benefits of self-harm, whilst acknowledging the risks. While the current study was not focussed on the process of stopping self-harm, other accounts of self-harm cessation suggest that a re-evaluation of the risks vs. the benefits may over time become a motivation for stopping (39, 40). There is also evidence that changes in life circumstances, or the social or environmental context in which self-harm occurs, may shift an individual's perceptions of the risks and benefits (41, 42). However, the current study suggests that emphasizing the risks of self-harm when individuals still perceive them to be outweighed by the benefits, or if they are ambivalent about the risks, may be ineffective and potentially counter-productive. Elsewhere, people who self-harm have indicated that they may respond better to interventions that acknowledge the need to manage rather than eradicate self-harm, for example through harm minimization strategies (22, 43).

### Limitations

The limitations of the study as a whole, including the different communication channels used for the interviews and the various lengths of time since participants' last self-harm, are discussed in Norman et al. (10). With regard to the current report, it should be noted that the original research question concerned the experience of self-harm among people who reported difficulties identifying and describing their feelings. It was not the original purpose of the study to explore the effect of other people's negative views of self-harm on those who engage in it. A study with that purpose might choose different questions for the interview and more explicitly discuss the question with participants. Alternatively, other analytical methods such as discourse analysis might be used to understand the way in which the social discourse surrounding self-harm affects the way in which participants themselves talk about it. Because the participants all scored highly on the TAS20 it is not possible to say whether their experiences and thoughts about self-harm could be generalized to a wider population. However, it is notable that the subthemes presented here found resonance in other qualitative studies of self-harm, with no such inclusion criteria.

## Conclusion

The findings presented here provide additional insights into the way in which people who self-harm have to navigate the prevailing negative perception of the behavior. Understanding the processes by which people who self-harm may simultaneously acknowledge and resist the idea of self-harm as “bad” is vital step toward the goal of reducing recurrence.

## Data Availability Statement

Due to the sensitive nature of this research, participants of this study were not asked for consent for their data to be made available to others for further research, so supporting data is not available.

## Ethics Statement

This study was reviewed and approved by Middlesex University Psychology Department Ethics Committee, 6 June 2018, reference 4083. The participants provided their written informed consent to take part in this study.

## Author Contributions

The study formed part of the lead author, HN's, doctoral thesis, supervised by the other three, named authors. HN conceived of and designed the study, with the support of the other authors. Material preparation, data collection, transcription, and analysis were performed by HN. LM independently reviewed one transcript. Themes were derived by HN and discussed with LM. The first draft of the manuscript was written by HN and all authors commented on previous versions of the manuscript. All authors read and approved the final manuscript.

## Conflict of Interest

The authors declare that the research was conducted in the absence of any commercial or financial relationships that could be construed as a potential conflict of interest.

## Publisher's Note

All claims expressed in this article are solely those of the authors and do not necessarily represent those of their affiliated organizations, or those of the publisher, the editors and the reviewers. Any product that may be evaluated in this article, or claim that may be made by its manufacturer, is not guaranteed or endorsed by the publisher.
